# Combining mTOR Inhibition with Radiation Improves Antitumor Activity in Bladder Cancer Cells *In Vitro* and *In Vivo*: A Novel Strategy for Treatment

**DOI:** 10.1371/journal.pone.0065257

**Published:** 2013-06-17

**Authors:** Roland Nassim, Jose Joao Mansure, Simone Chevalier, Fabio Cury, Wassim Kassouf

**Affiliations:** 1 McGill Urologic Oncology Research, Division of Urology, McGill University, Montreal, Quebec, Canada; 2 Radiation Oncology, Medical Physics Unit, McGill University Health Center and Research Institute, Montreal, Quebec, Canada; Henry Ford Health System, United States of America

## Abstract

**Purpose:**

Radiation therapy for invasive bladder cancer allows for organ preservation but toxicity and local control remain problematic. As such, improving efficacy of treatment requires radiosensitization of tumor cells. The aim of study is to investigate if the mammalian Target of Rapamycin (mTOR), a downstream kinase of the phosphatidylinositol 3-kinase (PI3K)/AKT survival pathway, may be a target for radiation sensitization.

**Experimental Design:**

Clonogenic assays were performed using 6 bladder cancer cell lines (UM-UC3, UM-UC5, UM-UC6, KU7, 253J-BV, and 253-JP) in order to examine the effects of ionizing radiation (IR) alone and in combination with RAD001, an mTOR inhibitor. Cell cycle analysis was performed using flow cytometry. *In vivo*, athymic mice were subcutaneously injected with 2 bladder cancer cell lines. Treatment response with RAD001 (1.5 mg/kg, daily), fractionated IR (total 9Gy = 3Gy×3), and combination of RAD001 and IR was followed over 4 weeks. Tumor weight was measured at experimental endpoint.

**Results:**

Clonogenic assays revealed that in all bladder cell lines tested, an additive effect was observed in the combined treatment when compared to either treatment alone. Our data indicates that this effect is due to arrest in both G1 and G2 phases of cell cycle when treatments are combined. Furthermore, our data show that this arrest is primarily regulated by changes in levels of cyclin D1, p27 and p21 following treatments. *In vivo*, a significant decrease in tumor weight was observed in the combined treatment compared to either treatment alone or control.

**Conclusions:**

Altering cell cycle by inhibiting the mTOR signaling pathway in combination with radiation have favorable outcomes and is a promising therapeutic modality for bladder cancer.

## Introduction

Bladder cancer is a very prevalent disease in North America. In 2012, 55,000 men and 18,000 women were diagnosed with bladder cancer; 1 in 5 men and 1 in 4 women will die from their disease [1]. . Radical cystectomy which consists of the complete removal of the bladder, remains the “gold standard” treatment for invasive bladder cancer [2]. Radiation therapy is an attractive alternative as it preserves the bladder and allows for normal urinary and sexual functions [3]. However, the lack of local control of the disease as well as the significant toxicity that is associated with radiation therapy remains problematic [4]–[6]. To improve efficacy, several clinical trials on organ-sparing management were carried out to test the effects of combined chemotherapy and radiation [7], [8]. However despite numerous efforts, chemoradiation studies remain associated with suboptimal local control of disease and decrease survival compared with radical surgery. As such, there is an imperative need to increase radiosensitization of bladder cancer to increase efficacy by improving local control of disease and allowing for dose reduction to decrease toxicity of radiation therapy.

A signaling molecule that is extremely attractive and has recently drawn much attention for targeted therapy is the mammalian target of rapamycin (mTOR). More specifically, mTOR is a downstream serine/threonine protein kinase of the phosphatidylinositol 3-kinase (PI3K)/AKT pathway which plays a critical role in oncogenesis [9], [10]. Deregulation of the PI3K/AKT/mTOR pathway generates a favourable oncogenic environment and has been documented in a variety of human tumours including bladder cancer [11]. mTOR inhibition became an active area of research to develop and test small inhibitory molecules such as rapamycin analogues -notably RAD001 (Everolimus, Novartis) and CCI-779 (Torisol, Wyeth) to treat diverse diseases, including cancer. Recently, the first successful Phase III clinical trial involving an mTOR inhibitor was realized in patients with advanced renal cell carcinoma, who experienced an improvement in overall survival [12]. We recently published the first report demonstrating significant antitumor activity *via* inhibiting mTOR with RAD001 in bladder cancer models *in vitro* and *in vivo* [13]. Interestingly, remarkable differences in sensitivity to mTOR inhibition were noted among nine human bladder cancer cell lines. Moreover, there was no correlation between activated AKT and mTOR levels with cell aggressive features. However, this was not the case for activated S6 whose levels appeared higher in RAD001 sensitive compared to relatively resistant cell lines. Of interest, some studies have reported that mTOR inhibition may sensitize tumors of the prostate, breast, and brain to ionizing radiation [14]–[16]. Since radiation was shown to activate the PI3K/Akt survival/growth pathway which may be responsible for the cell death escape and radioresistance [17], [18], concurrent mTOR inhibition may potentially overcome resistance to radiation in bladder cancer. To follow up on this hypothesis, the present study examined the effects of combining RAD001 and ionizing radiation, *in vitro* and *in vivo*, on cell survival and growth in an array of bladder cancer cell lines. In addition, we attempted to shed light on the mechanism by which this combination of treatments might inhibit tumor growth.

## Materials and Methods

### Ethics Statement

All ethical standards associated with the use of our animal xenograft model were fully followed and respected. The McGill University Health Center's Facility Animal Care Committee approved our animal protocols (protocol #5428) before the beginning of the study. Furthermore, the animals were maintained and kept in state-of-the-art facilities that follow the stringent procedures for conducting animal research, which includes constant monitoring and inspection of the animals and the users.

### Cell culture

The UM-UC3, UM-UC5, UM-UC6, and KU7 cell lines were characterized and provided by the Specimen Core of the Genitourinary Specialized Programs of Research Excellence in bladder cancer at M. D. Anderson Cancer Center [19]. The 253-JP and 253J-BV were kindly provided by Dr Colin P.N. Dinney from M.D. Anderson Cancer Center, Houston, Texas [20]. The cell lines were routinely cultured at 37°C in a 5% CO_2_ incubator, maintained in Eagle's minimum essential medium (EMEM) containing 10% fetal bovine serum (FBS) (Wisent, Saint-Jean-Baptiste QC) and passaged when reaching 80% confluence. The mTOR inhibitor RAD001 was kindly provided by its manufacturer, Novartis.

### Clonogenic assay

Cells were seeded in a 6-well plate at a density of 200 cells per well and maintained in the growth medium. Once attached, they were treated with RAD001 at doses equivalent to the GI50 for each cell line, as previously described [13]: UM-UC3 (75 nM), KU7 (50 nM), 253J-BV (8 nM), 253-JP (8 nM), UM-UC5 (0.5 nM) and UM-UC6 (0.5 nM) and maintained at 37°C in a 5% CO_2_ incubator for 12 hours. This was followed by radiation treatment at different dosages, with and without RAD001. Controls included untreated cells along with cells treated with each of radiation and RAD001 treatment alone. Cells were further cultured at 37°C and allowed to form colonies for 10–14 days. An approximate cutoff of 50 viable cells/colony was chosen. The cells were washed with phosphate balanced salt solution (PBS) and fixed for 15 min using 3.7% formaldehyde in PBS. After a second PBS wash, cells were stained with crystal violet (0.4% w/v in PBS; Fisher Scientific, Waltham, MA) and left to air dry before counting of colonies. Each treatment consisted of duplicate wells of a 6-well plate and the experiment was performed twice. The surviving fraction was calculated as (the mean colony count at the end of the experiment)/(cells inoculated at the beginning)×(plating efficiency). The plating efficiency was defined as (mean colony count)/(cells plated in the non-radiated control). The non-irradiated cells were used as a control.

### Flow cytometry

Cells were seeded in culture plates and allowed to attach. RAD001 was added to the appropriate samples 12 hours before radiation at a dose equivalent to the GI50 of each cell line. This was followed by a dose of 4Gy of ionizing radiation (based on previously determined sensitivity experiments) and the cells were further cultured for 48 hours. Cells were then trypsinized, washed once with PBS, and fixed with 100% cold ethanol for 60 minutes at 4°C. After centrifugation, cell pellets were resuspended in a solution of propidium iodide (PI) (50 g/ml, Invitrogen, Carlsbad, CA) in PBS, supplemented with RNase (100 g/ml; Invitrogen, Carlsbad, CA) then transferred to fluorescence-activated cell sorting (FACS) tubes and incubated in the dark for 30 min at 40°C to allow propidium iodide intake in the nucleus. PI intake was then assessed using a Coulter Flow Cytometer (BD Biosciences, Franklin, NJ).

### Western blot

Cells were grown and treated as per the regimen described above (RAD001, ionizing radiation, and both in combination), with untreated cells serving as controls. Following treatments, cells were scraped on ice and re-suspended for 30 minutes at 4°C in cold RIPA (lysis) buffer containing a cocktail of phosphatase and protease inhibitors (Roche Diagnostics, Indianapolis, IN). Cell suspensions were then centrifuged to collect clear lysates in the supernatant. The protein concentration was measured by the bicinchoninic acid (BCA) assay (Pierce Scientific-Thermo Fisher Scientific, Rockford, IL). Protein samples (40 µg–60 µg) were submitted to polyacrylamide gel electrophoresis, as previously described [13]. Proteins in gels were transferred onto membranes, blocked with a 5% non-fat milk and/or 5% bovine serum albumin solution, and immuno-blotted with the following monoclonal primary antibodies (all rabbit): phospho-AKT, total AKT, phospho S6, total S6, p21, p27kip1, and cyclin D1 (Cell Signaling Technology, Beverly, MA) at concentrations recommended by the manufacturer. The membranes were then incubated with the appropriate anti-rabbit secondary antibodies and an ECL chemiluminescence detection system (Amersham-GE Healthcare, Piscataway, NJ) was used to reveal protein bands of interests on X-ray film. The films were scanned and protein levels were normalized against actin (Cell Signaling Technology, Beverly, MA), a control 42 kDa housekeeping protein present in all samples and served as our loading control.

### In vivo—Xenograft model

All protocol approvals were obtained prior to the onset of the study from the Animal Care Committee of the McGill University Health Center. Female athymic mice (Nu/Nu strain, 4–6 weeks old; Charles River Laboratories, Wilmington, MA) were used for our xenograft bladder cancer model, as previously reported [13]. Briefly, mice were subcutaneously injected with KU7 (10^6^ cells per injection). Another experiment with the same methodology was performed using the 253J-BV bladder cancer cell lines. To facilitate adhesion, cells were suspended in 200 µl Matrigel (BD Biosciences, Franklin, NJ) prior to injection. Tumors were allowed to implant and grow for one week prior randomization into 4 groups corresponding to the different treatment arms, with each group consisting of 14 mice. The 1^st^ group was treated with a placebo (5% glucose solution in water). The 2^nd^ group received RAD001 orally (microemulsion diluted in 5% glucose solution) at a dose of 1.5 mg/kg daily. In the 3^rd^ group, tumors were exposed to ionizing radiation at a fractionated dosage totaling 9 Gy (3×3Gy) every second day during the first week of treatment. In the 4^th^ group, mice were given RAD001 at the above-mentioned dosage 1 day before the start of the tumor radiation treatment. Mice were followed for 4 weeks from the onset of treatments. Body weight and animal behavior were monitored throughout the experiment. Tumors were measured (length and width) twice a week using a Vernier caliper in order to calculate volumes (V = [(length×width^2^)×(π/6)] as previously reported [13]. Mice were euthanized in a CO_2_ chamber at the end of treatment. Tumors were harvested, immediately weighed, and conserved fixed or frozen for future studies.

### Immunohistochemistry

Tumor sections were obtained from mice treated with placebo, radiation, RAD001 and the combination regimen. The paraffin embedded tumors sections were mounted on glass slide for staining. Following de-paraffinization and hydration, antigen retrieval was performed in heating the samples with 5% citrate buffer solution (pH 7.0). The sections were incubated overnight at 4°C with a p21 specific antibody (dilution 1∶25). HRP-conjugated goat polyclonal anti-rabbit IgG secondary antibody was added and incubated at room temperature for 1 hour. 3,3′-Diaminobenzidine (DAB) substrate (Sigma Aldrich, St. Louis, MO) was used for color development according to manufacturer's instructions. Slides were viewed under a Leica Diaplan inverted microscope equipped with a Leica DFC300FX Camera (Leica, Wetzlar, Germany). Pictures were captured using a Leica Application Suite. Analysis was based on an average of 5 foci, at 40× magnification, showing viable cells, and a computed H-score was calculated by summing the products of the percentage cells stained at a given staining intensity (0–100) and the staining intensity (0 for negative staining, 1 for low and 2 for high staining).

### Statistical analysis

Student's T-test (unpaired, two-tailed) was used in all statistical analysis. Significance was set at p<0.05.

## Results

### Relative sensitivity of a panel of bladder cancer cell lines to RAD001 and ionizing radiation

**Figure 1:**
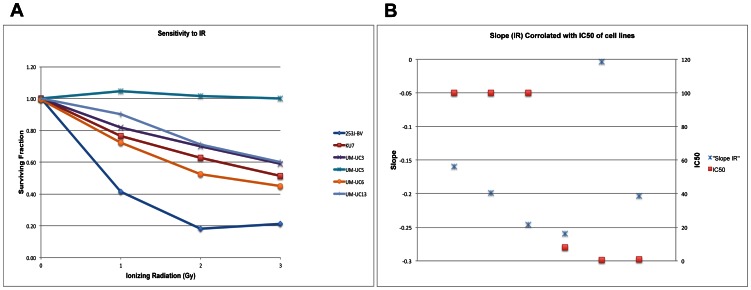
Response of a panel of bladder cancer cell lines to ionizing radiation. Plated cells were exposed to ionizing radiation to measure the effects on growth by clonogenic assay, as described in Methods. (**A**) Based on the gathered results, we were able to classify these cell lines as radiation–sensitive, moderately sensitive and -relatively resistant. (**B**) The RAD001 IC50 was plotted against the slope of the curve for each cell line in the clonogenic assay when treated with IR.

We recently demonstrated that a panel of nine bladder cancer cell lines exhibits relative differences in their RAD001 sensitivity and accordingly, RAD001 treatment resulted in relative differences in mTOR inhibition and growth arrest, as monitored by MTT assays. With this data, we were able to divide our cells lines into 3 groups based on their RAD001 sensitivity [13] as follows: relatively resistant (UM-UC3, UM-UC13, KU7 (GI50≥50 nmol/L)), moderately sensitive (253J-P, 253J-BV, RT4 (GI50<50 nmol/L)) and finally highly sensitive (UM-UC1, UM-UC5, UM-UC6 (GI50≤0.5 nmol/L). In this study looking at the effects of combined treatments (RAD001 and radiation), clonogenic assays was used to classify the six cell lines tested according to their relative sensitivities to IR to various doses of radiation (Fig. 1A). Based on these relative sensitivities to radiation, cell lines were divided into three groups, resistant, moderately resistant, and sensitive. The resistant group includes UM-UC5 with the highest surviving fraction, the moderately resistant included UM-UC13, KU7, UM-UC3, UM-UC6 whereas 253J-BV had a lower surviving fraction and was therefore defined as a radiation-sensitive cell line. We compared the response of these six cell lines to each of RAD001 and ionizing radiation. Based on the data in Figure 1B and Table 1, we concluded that there is no correlation between the sensitivity to RAD001 and the sensitivity to ionizing radiation.

**Table 1:** Classification of bladder cancer cell lines based on their relative response to RAD001 and ionizing radiation.

	Ionizing Radiation			RAD001		
Cell Line	Sensitive	Moderately Resistant	Relatively Resistant	Sensitive	Moderately Sensitive	Relatively Resistant
*253J-BV*	**x**				**x**	
*KU7*		**x**				**x**
*UM-UC3*		**x**				**x**
*UM-UC5*			**x**	**x**		
*UM-UC6*		**x**		**x**		
*UM-UC13*		**x**				**x**

No correlation was noted when the RAD001 response, as reported [13], was compared to the response to ionizing radiation.

### Ionizing radiation activates AKT while RAD001 inhibits S6 phosphorylation

**Figure 2:**
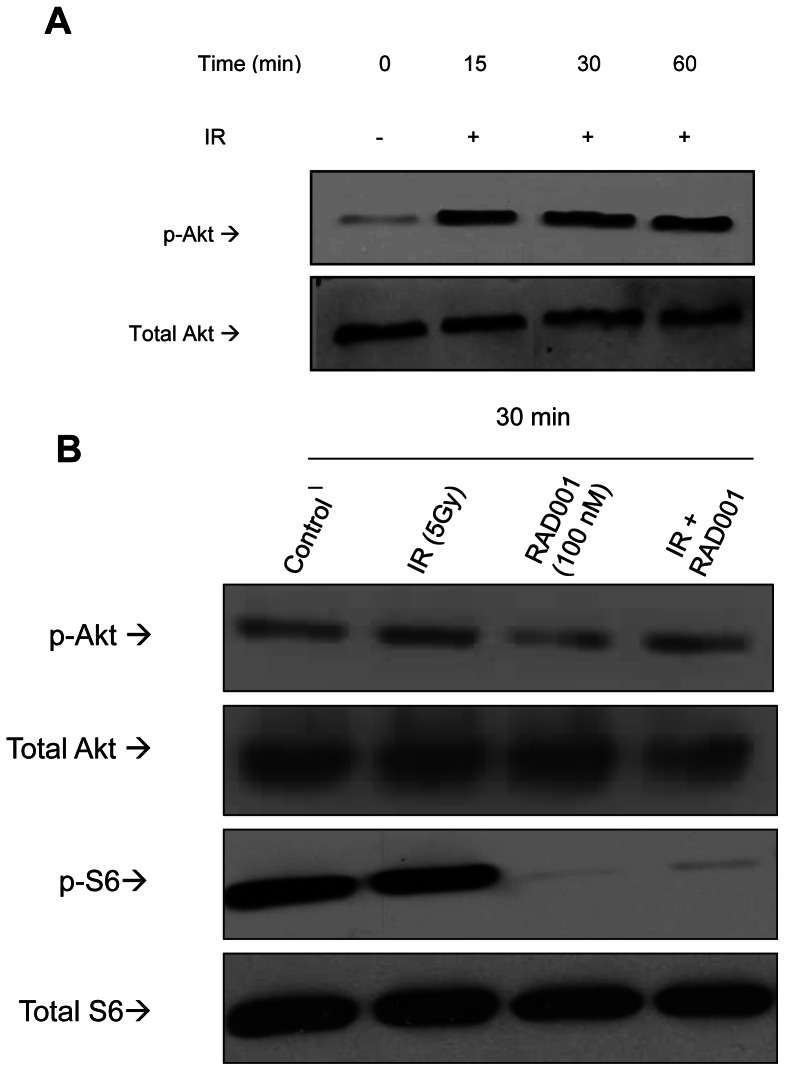
Ionizing radiation activates AKT by phosphorylation and RAD001 inhibits S6 phosphorylation. (**A**) KU7 cells were treated with 4 Gy of ionizing radiation. Cells were lysed 15, 30 and 60 minutes following treatment to analyze AKT activation (p-AKT; upper row) by Western blot. Total levels of AKT are shown in the lower row. (**B**) KU7 cells were treated with 5 Gy of radiation, 100 nM of RAD001 or both, and lysed. Levels of Akt and S6 phosphorylation were analyzed by Western blot. Total levels of Akt and S6 expression are shown. Similar results were obtained when 253J-BV, UM-UC3, and UM-UC6 were treated with radiation and RAD001 alone and in combination (data not shown).

It has been reported that ionizing radiation activates AKT in the surviving cell fraction [17], [21]. As this may be associated with resistance to treatment, cell death escape and survival, we sought to determine if radiation exposure of bladder cancer cells would lead to AKT activation. For this purpose, a relatively resistant cell line, KU7, was exposed to ionizing radiation over time (0 to 60 min) and lysed to analyze pAKT by direct Western blotting using phospho-specific AKT antibodies directed against the S473 phosphorylation site. Results in Figure 2A show that indeed AKT was rapidly activated following 15 min of radiation treatment and this activation persisted at 30 and 60 min. These results thus imply that KU7 undergo an activation of the pro-oncogenic survival pathway following exposure to ionizing radiation. In all experiments, the levels of pAkt increased following the treatment with ionizing radiation to a maximum and decreased afterwards. Similarly, as KU7 cells are also relatively RAD001 resistant, they were treated with RAD001 to ascertain that its target mTOR was inhibited. For this purpose, levels of phosphorylated S6 were determined using an antibody specific to serine residues 240/244 in the S6 protein. As expected, RAD001 was potent in decreasing phosphorylation levels on the mTOR downstream signaling molecule and target S6, as shown at 30 minutes post-treatment (Fig. 2B) and this inhibition is sustained at 24 h post-treatment (data not shown). Furthermore, similar results were obtained with other bladder cancer cells lines (253J-BV, UM-UC3, and UM-UC6) treated with radiation and RAD001 (data not shown).

### Combining RAD001 with ionizing radiation significantly reduces colony formation

**Figure 3:**
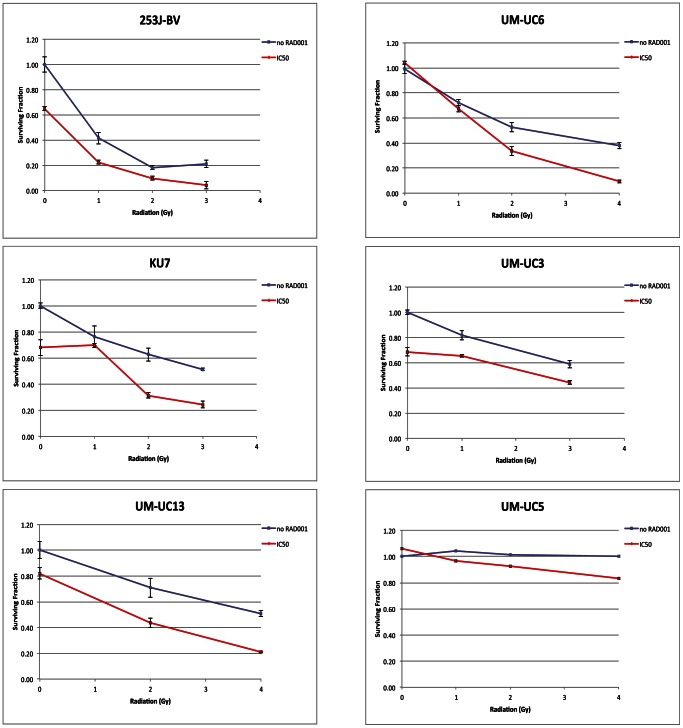
Effect of RAD001 and ionizing radiation on colony formation. Six cell lines were treated with RAD001 for 12 hours before exposure to ionizing radiation and further grown as indicated in Methods. Colony formation was measured after cell fixation and staining with crystal violet, 10–14 days after treatment depending on cell lines. Results were statistically significant (p<0.05) in the combined treatment compared to either treatment alone in all tested cell lines.

To provide insight on effects of combining RAD001 with ionizing radiation on bladder cancer cell lines, we monitored the fraction of surviving cells over time using clonogenic assays. Following treatment, plated cells were monitored over time and the number of colonies was counted. The RAD001 dose was maintained at the GI50 for each cell line, while the radiation dose was varied. In all cell lines tested (253J-BV, UM-UC6, KU7, UM-UC3, UM-UC13, and UM-UC5), a significant decrease in the number of colonies was observed for cells treated with the combination therapy compared to either ionizing radiation alone, or the untreated control (Fig. 3). Interestingly, while this decrease in the surviving fraction was seen in all cell lines tested, the most dramatic relative decrease when both treatments were combined was seen with two most sensitive cell lines to RAD001 (UM-UC5 and UM-UC6). In all tested cell lines, our results point to an additive effect on growth when combining RAD001 with ionizing radiation. It is worth noting that a lower inhibition of colonic formation was observed in the two cells lines (UM-UC5 and UM-UC6) that were characterized originally by our laboratory to being the most sensitive to RAD001. This lies primarily with the colonogenic assay itself where colonic formation (as determined by a universally set colony size) whereas the sensitivity to RAD001 was done with an enzymatic assay (MTT). This discrepancy in sensitivities of the assays, length of assay, combined with the quick doubling time, could explain the clonogenic results for these two cells lines.

### The treatment with RAD001 and ionizing radiation induces both an increase of the percentage of cells in the G0/G1 and the G2 phases of the cell cycle

**Figure 4:**
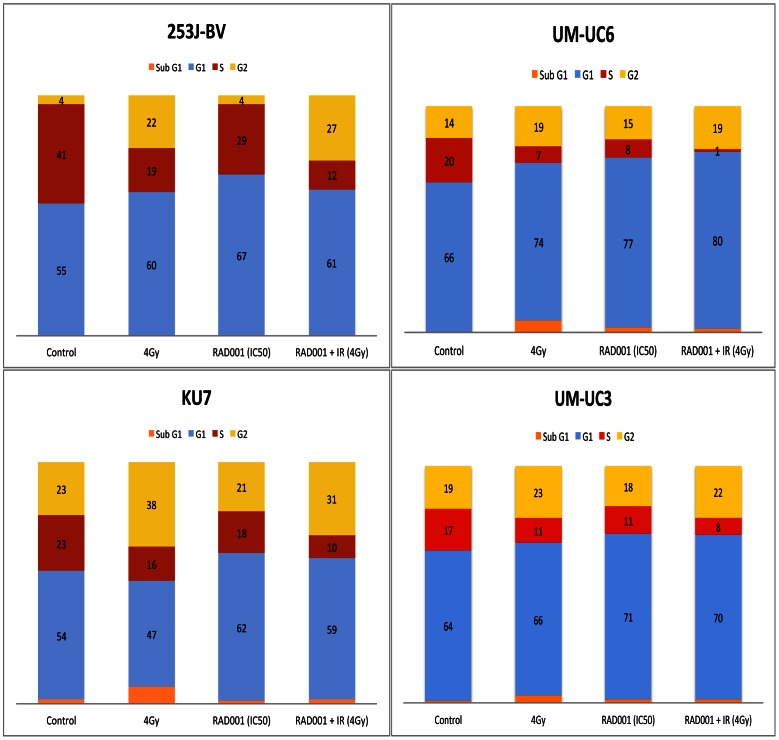
Effect of RAD001 and ionizing radiation on the cell cycle. The cell lines were cultured and treated with RAD001 alone, ionizing radiation alone and with the combination of RAD001 and radiation. For the latter series, samples were pre-treated with RAD001 for 6 hours prior to radiation. Cells were fixed and stained for propidium iodide intake at 48 hrs after treatment, and then measurements were performed by flow cytometry. The proportion of cell populations in the different phases of the cell cycle is shown for each cell line by colored bars (G0/G1: Orange/Blue; S: Red and G2: Yellow).

To get insights into the mechanism underlying the observed growth inhibition, cell cycle analysis was performed by flow cytometry to study the distribution of cells throughout the various phases of the cell cycle 48 hours following each treatment alone and in combination. The cells were treated with a dose of RAD001 equivalent to their GI50 (ranging from 0.5 to 75 nmol/L) as well as 4Gy of ionizing radiation. Results are shown in Figure 4. RAD001 induced a G0/G1 arrest in all the bladder cancer cell lines tested: KU7 62%±4%, UM-UC3 71%±6%, UM-UC6 77%±3% and 253J-BV 67%±4% compared to their untreated controls, 54%±3%, 64%±2%, 66%±2% and 55%±3%, respectively. Percentages represent the ratio of cells in each phase relative to the total number of cells. As expected, ionizing radiation led primarily to a G2 arrest, illustrated by a significant increase in the percentage of cells in this phase following treatment with ionizing radiation: KU7 38%±4%, UM-UC3 23%±4%, UM-UC6 19%±4% and 253J-BV 22%±3% compared to their respective untreated controls: 23%±2%, 19%±3%, 14%±3% and 4%±2%, respectively. In the combined arm with RAD001 and ionizing radiation, we observed both an increase in the percentage of cells in G0/G1 and G2 phases (Fig. 4). More specifically, a decrease in the percentage of cells in the S-phase was observed compared to either treatment alone or to the control (no treatment) and this was paralleled with an increase of the percentage of cells in the G0/G1 and the G2 phases. Taken together, we concluded that the cytostatic effect of RAD001 combined with ionizing radiation exhibits an inhibitory additive effect on the progression of cells through their cycle.

### RAD001 and ionizing radiation alter the levels of the cell cycle checkpoints cyclin D1, p27kip1 and p21

**Figure 5:**
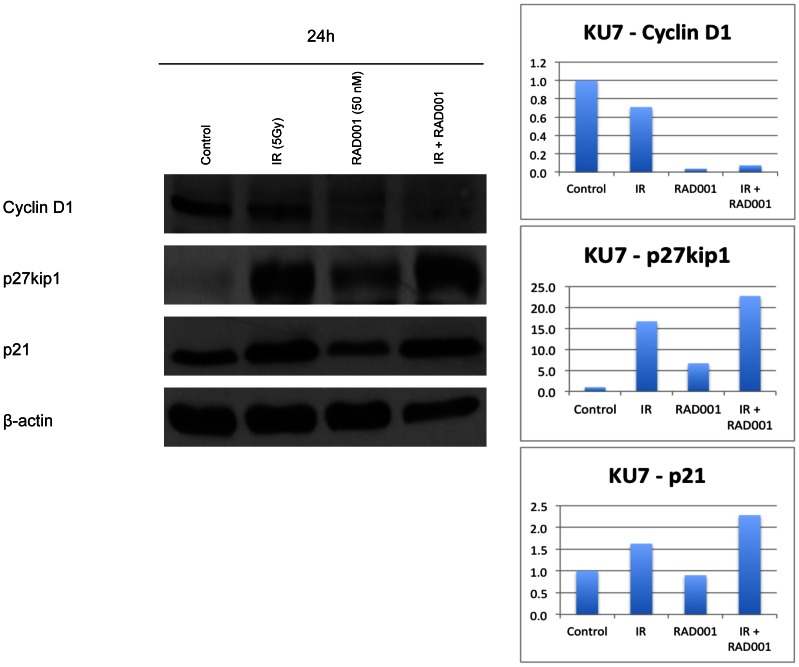
Expression of cell cycle regulatory proteins following RAD001 and IR treatment. Bladder cancer cells were treated with RAD001, ionizing radiation (IR) or the combined treatment. They were lysed 24 hours after treatment as described in Methods. Western blot analysis for Cyclin D1, p27kip1 and p21 in KU7 cells and normalized in lower panels as a function of the actin level measured in parallel. Similar results were obtained for all cell lines tested, UM-UC3, UM-UC6 and 253J-BV (not shown).

Based on the above cell cycle analysis data and to further understand the mechanism by which RAD001 and ionizing radiation act together to inhibit cell growth, we tested the likelihood of changes in expression levels of diverse regulators of the cell cycle, particularly associated with checkpoints such as cyclin D1, p27, and p21. Results in Figure 5 illustrate the case of KU7, used as a representative of the group of cell lines found to be relatively resistant to both ionizing radiation and RAD001. That being said, a dose of RAD001 equivalent to the GI50 of that cell line was used to ensure a response to the RAD001 treatment. Our results show that the level of cyclin D1, which is a protein required for the G1/S transition through the cell cycle, was decreased following 24 hours of treatment with RAD001 (Fig. 5), a finding that supports our observations on cell cycle changes. In contrast, levels of p27, which is a protein also associated with the G1/S transition, changed in an inverse correlation (increased) following 24 hours of treatment with RAD001, ionizing radiation, and the combined treatment compared to the control (Fig. 5). Interestingly, levels of p21, which is also associated with inhibiting cell cycle progression, were decreased in cells treated with RAD001 alone compared to control, or radiation (Fig. 5). The levels of p21 increased in response to the treatment to radiation compared to the control, and the levels of p21 are at their highest when cells are treated with both RAD001 and ionizing radiation. It is noteworthy that similar results were obtained for cyclin D1, p27, and p21, in all tested cell lines: UM-UC3, UM-UC6 and 253J-BV.

### Combining RAD001 treatment with ionizing radiation significantly inhibits tumor growth in human bladder cancer xenograft model, compared to either treatment alone

**Figure 6:**
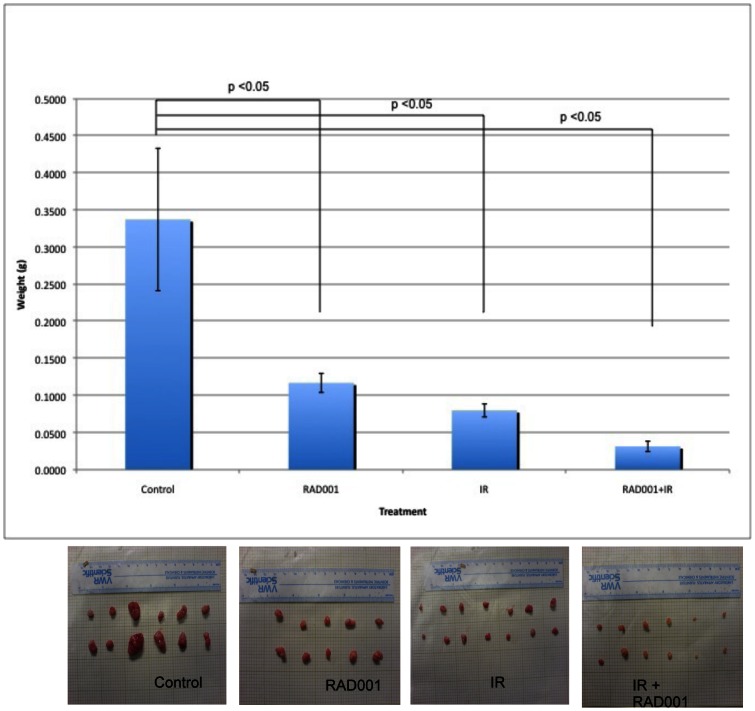
Effect of RAD001 and ionizing radiation on bladder cancer tumor weight *in vivo*. The athymic mice bladder tumor model of KU7 was used as described in Methods. Tumor weights (in grams) reached at the experimental endpoint and expressed as mean weight of tumors harvested for each group of mice in the 4 treatment arms, as indicated. Similar findings obtained using 253J-BV cells (data not shown).

**Figure 7:**
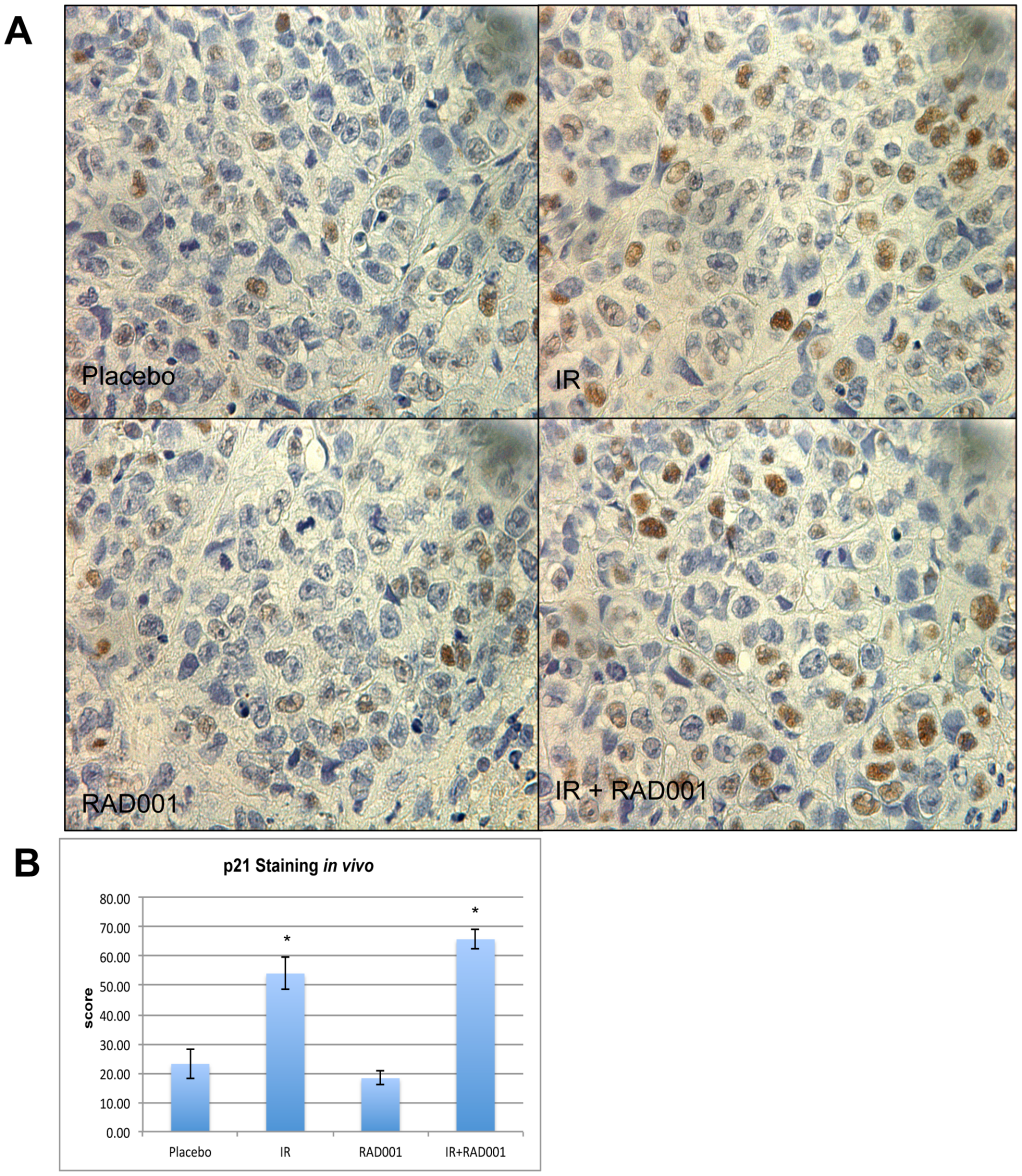
Immunohistochemical p21 levels in mouse xenograft paraffin sections. (**A**) Immunohistochemistry was used to detect the levels of p21 in paraffin-embedded mouse xenograft bladder cancer tissues treated with placebo, IR, RAD001 and in combination. (**B**) Quantification of the immunohistochemistry data revealed a significant increase in p21 expression as observed in tumors treated with ionizing radiation and in combination compared to the placebo and RAD001 treatment.

To ascertain significance and to verify if *in vitro* data with regards to effects of combining RAD001 and ionizing radiation on growth of bladder cancer cell lines can be transposed *in vivo*, we used the KU7 bladder cancer cell line to grow as subcutaneous tumor xenografts in athymic mice. In all the mice, tumors were evidenced within the first 10 days after implantation. There was no body weight loss or any significant toxicity directly associated with RAD001 and ionizing radiation treatments during the entire duration of the study (a total of 5 weeks). In mice treated with combined RAD001 and ionizing radiation, there was a maximal inhibitory effect on bladder cancer progression, as indicated by the significant decrease in tumor weight compared to either treatment alone or placebo group (average tumor weight 31 mg for combination arm vs. 117 mg with RAD001 alone, 80 mg with IR, or 340 mg for placebo, P<0.05) (Fig. 6). Similar findings were also obtained using the 253J-BV cell lines where combined therapy achieved maximal inhibitory effect on bladder cancer progression after 4 weeks of treatment compared to control. The same results were demonstrated using tumor growth kinetics when tumor volume was measured throughout the treatment duration (data not shown). Our p21 immunohistochemistry staining on the xenograft sections confirmed our findings from the western blot analysis for p21 levels *in vitro* (Fig. 7). The levels of p21 significantly (p<0.05) increased following the treatment with radiation and the combination regimen when compared to the placebo. Furthermore our data indicated a slight decrease, although statistically non-significant, of the p21 levels in the RAD001-treated group alone.

## Discussion

Radiation therapy is a key element of many cancer treatment regimens hence its widespread use. However, ionizing radiation appears to contribute to an unfavorable increase in signaling through the PI3K/AKT/mTOR pro-survival pathway. In the present study, we observed differences in the sensitivity of a panel of six bladder cell lines to ionizing radiation, with some being were more resistant than others. We also demonstrated the activation of AKT following exposure to ionizing radiation. Several factors may potentially be determinant in the activation mechanisms of the PI3K/AKT pathway following ionizing radiation and then help cancer cells in the establishment of resistance [22]. Among others, the enhanced activity of key enzymes such as telomerase activity [23] as well as the involvement of signaling molecules such as the epidermal growth factor receptor (EGFR) and RAS [24], [25] may explain why some tumors do not respond to radiation as effectively as others. Notably, EGFR signaling through the PI3K/AKT was reported to regulate the DNA-dependent protein kinase catalytic subunits, which are part of the DNA repair machinery turned on following radiation [26]. While these observations emphasize the important role that the activation of PI3K/AKT plays in the cancer radioresistance, we demonstrate that blocking the PI3K/AKT/mTOR pathway with RAD001 appears as a valuable mean to enhance the efficacy of radiation treatment in bladder cancer cells. The mechanism by which RAD001 exhibits this enhanced effect still needs further evaluation. It could simply be that blocking the rebound activation of the pathway following radiation is sufficient to decrease radioresistance; a plausible mechanism as the two treatments do share common targets in the cell such as the hypoxia inducible transcription factor (HIF-1), a molecule downstream of mTOR [27].

In addition to cellular signaling, the efficacy of the treatments may lie on their effects on the cell cycle. Our analyses show that RAD001 induces a G0/G1 arrest in the cells while ionizing radiation induces an S/G2 arrest. In the combined therapy, we observe both a G0/G1 as well as a G2 arrest. These changes in the cellular population within each part of the cycle were compensated by a decrease of cells in the S-phase. Since the early 1960s, scientists have confirmed that the sensitivity to radiation is dependent upon the phase of the cell cycle whereas cells are most sensitive to radiation in the late G1 and the G2/M phase, and are least sensitive in the S Phase [28]–[30]. These responses include chromosome aberrations, delay in division, alterations in DNA division and survival [31]. Our findings indicate that shifting cells within the phases of the cell cycle, following the treatment with RAD001, and arresting them in specific phases will alter their sensitivity to ionizing radiation. Furthermore, our preliminary data indicate that when the cells are examined at 12 h post-radiation treatment, a more dramatic shift to the G2 phase occur in the combination treatment as opposed to radiation alone, potentially rendering them more sensitive to IR. In our experiments, cells were arrested at the G1 phase with decreased proportion of cells in the S-phase following the pre-treatment with RAD001, and this arrest is rendering the cells more sensitive to ionizing radiation. It would be interesting to examine whether the effects of the combination in the regimen consisting of fractionated doses rather than a single dose will further increase the efficacy of RAD001 in addition to radiation in bladder cancer cells.

The cycle arrest induced following the treatment alone or in combination is underlined by changes in the levels of various proteins that control passage through the phases and the progression of the cycle. Here, we report a decrease in cyclin D1 levels following treatment with RAD001 alone and when combined with radiation. A decrease in cyclin D1 results in the lack of cyclin D1/cdk complex formation required for transition past the G1 phase. Furthermore, our report points towards an increase in p27kip1 when treated with RAD001 and a maximal effect is observed in the combined treatments. This increase in the p27kip1 expression levels, which is an inhibitor of cyclin D1/cdk4 complex, support involvement of inhibitors of the cell cycle in the mechanism by which these treatments alter cellular proliferation [32]–[35].

Although the entire mechanism for the inhibitory role that RAD001 and ionizing radiation exhibit on the cell cycle remains unclear, one important protein that needs to be studied closely with that regards is the cyclin dependant kinase inhibitor 1, p21. It has been reported that the rapamycin-induced disruption of the cdk2 interaction with PCNA was due to the down regulation of p21, which affects the interaction between cdk2 and cyclin D1, leading to the malformation of the complex required to move the cells past the G1 phase [34]. Aside from being a G1/S regulatory molecule, p21 is also involved in DNA damage repair following exposure to ionizing radiation. Our results indicate that levels of p21 decrease when treated with RAD001 alone and increase following the treatment with ionizing radiation. *In vitro* and *in vivo*, p21 levels were maximally elevated in the combination arm, pointing towards the involvement of p21 in the increased cell cycle arrest observed previously by flow cytometry. It has been shown that p21 interacts with PCNA [36], the proliferating cell nuclear antigen. This p21/PCNA interaction has an inhibitory effect on DNA synthesis, a major process in DNA damage repair, and subsequently leading to an arrest in the G2/M phase [37], [38]. Hence, pretreatment with RAD001 can enhance the effects of radiation through alteration in p21 levels that affects DNA damage repair leading cells to further arrest in G2. This may seem contradictory to the inhibitory role of p21, but studies have shown that p21 may exhibit a cell proliferation role [39]. p21 may exist at an optimal level in the cells and that a certain fluctuation from the basal level can lead to its inhibitory effect. Of note, p21 activity can also be influenced by its state of phosphorylation and location within the cell [40].

Another key element that might be playing an important role in determining the response of the cells to radiation and the effects on cell cycle is the tumor suppressor p53. P53 is an important DNA damage checkpoint that was shown to be involved in either a cell cycle arrest or apoptosis depending on the levels of p53 in the cell and the status of the p53 gene [41]. That being said, it has been demonstrated that the activation of p53 results in the activation of p21 leading to cell cycle arrest [42]. In our tested cell lines, we noticed higher baseline levels of p21 in cells that are p53-WT (253J-BV and UM-UC6) compared to p53-mutant cells (KU7 and UM-UC3) (data not shown). Surprisingly, 253J-BV and UM-UC6 had a significantly lower GI50 for RAD001 compared to KU7 and UM-UC3. While the mechanism of actions of RAD001 and ionizing radiation together is not fully understood, these observations indicate a possible cross talk between the p53/p21 pathway (which is activated by radiation) and the mTOR pathway.

Our *in vitro* results seemed to be well echoed in our *in vivo* xenograft model where we report a significantly slower growth rate that translated into smaller tumor weights (p<0.05) observed at the end of treatment in all treated groups compared to the untreated group. More interestingly, we observed the lowest tumor weights (p<0.05) in the group treated with the combination arm of RAD001 and ionizing radiation compared to all other groups. When untreated, the tumors grew at a much faster pace and weighed more than tumors of mice treated with RAD001 and ionizing radiation alone.

Our study shows clearly that RAD001, alone and in combination of radiation therapy, exhibits a cytostatic effect on tumor cells. Our previously published report showed also that no apoptosis is induced following the treatment of RAD001 alone when measured by propidium iodide uptake. Of interest, we have remarked an induction of autophagy as measured by levels of the autophagic marker, the light chain 3A (LC3) protein, in the cells following the treatment with RAD001 and in combination with ionizing radiation. Future research in our laboratory will further evaluate other types of cell death in the combination arm including autophagy and mitotic catastrophe.

## Conclusion

To our knowledge, this is the first report of an enhance effect when combining RAD001 with radiation in bladder cancer *in vitro* and *in vivo*. The proposed treatment regimen is very promising and may potentially provide a remarkable advancement in the management of bladder cancer to improve clinical outcomes. These findings formed a platform on which a phase II homegrown clinical trial evaluating RAD001 combined with chemoradiation is now open at the McGill University Health Center targeting patients with invasive bladder cancer.
